# Language Experience Influences Sociolinguistic Development: The Role of Speaker Race and Language Attitudes on Bilingual and Monolingual Adults’ Accent Processing

**DOI:** 10.3390/brainsci14101028

**Published:** 2024-10-17

**Authors:** Vanessa Ritsema, Rebeka Workye, Drew Weatherhead

**Affiliations:** Department of Psychology and Neuroscience, Dalhousie University, 6299 South St, Halifax, NS B3H 4R2, Canada; vn268273@dal.ca (V.R.); rebeka@dal.ca (R.W.)

**Keywords:** accent intelligibility, bilingualism, sociolinguistics, psycholinguistics, speech perception

## Abstract

Background/Objectives: Speaker race and the listener’s language experience (i.e., monolinguals vs. bilinguals) have both been shown to influence accent intelligibility independently. Speaker race specifically is thought to be informed by learned experiences (exemplar model) or individual biases and attitudes (bias-based model). The current study investigates speaker race and the listener’s language experience simultaneously as well as listeners’ attitudes toward non-native speakers and their ability to identify the accent. Methods: Overall, 140 White English monolinguals and 140 English/Norwegian bilinguals transcribed 60 Mandarin-accented English sentences presented in noise in the context of a White or East Asian face. Following sentence transcription, participants were asked to rate the strength of the accent heard and completed a short questionnaire that assessed their accent identification ability and their language usage, proficiency, familiarity, and attitudes. Results: Results show that a listeners’ ability to identify an accent and their attitudes toward non-native speakers had a significant impact on accent intelligibility and accentedness ratings. Speaker race by itself did not play a role in accent intelligibility and accentedness ratings; however, we found evidence that speaker race interacted with participants’ accent identification scores and attitudes toward non-native speakers, and these interactions differed as a function of language experience. Conclusions: Our results suggest that bilinguals’ sociolinguistic processing may be more in line with a bias-based model than monolinguals.

## 1. Introduction

Accented speech conveys a considerable amount of socially meaningful information, playing an essential role in the categorization of speakers into social groups [1,2,3]. Despite the wide range of information needed to be processed, listeners are strikingly sensitive to even slight phonetic differences in speech with research suggesting an ability to detect a foreign accent in speech segments as short as 30 ms [4]. Accent intelligibility is becoming increasingly relevant in a period of unprecedented globalization and immigration [5], and increased exposure to foreign accents becomes inevitable. Furthermore, the underlying mechanisms behind accented speech processing is of particular concern given the communication issues and discrimination often faced by speakers with non-native accents [6,7,8].

The intelligibility of accented speech is impacted by both the acoustic properties of speech and the social information of the speaker [2,9,10,11,12]. Of interest to the current study is speaker race, which is indexed by visual information provided by a speaker’s face. Many studies have demonstrated that speaker race influences how well accented speech is recognized and comprehended, although there is conflicting evidence regarding how race influences comprehension and intelligibility [10,13,14,15,16,17]. Conflicting findings for the effects of speaker race can be contextualized by two prominent theories on race and accent intelligibility: the bias-based framework and the exemplar framework.

The bias-based framework claims that perceiving a speaker as non-native activates biases and stereotypes causing the listener to exert less effort in understanding the speaker, resulting in weakened comprehension [16]. Offering some support for this framework, research in intergroup relations suggests correlations between foreign accents, out-group categorization, and negative perceptions [3,6,8]. Using matched-guise designs, numerous studies report that leading listeners to believe they will hear a non-native speaker using nationality labels or different faces often result in stronger accent ratings and weakened accent comprehension, regardless of whether the speaker is non-native [16,17,18].

In contrast, in an exemplar framework, listeners use social knowledge to predict and interpret speech [10]. Once primed with a specific social cue about a speaker, there is increased activation of stored episodic traces that are linked with said social cue, such as specific accent associations, which aid in speech intelligibility [19]. McGowan [10] demonstrated this effect by showing that American listeners better comprehended Mandarin accented English when it was paired with an East Asian face as opposed to a White face. Similarly, event-related potential (ERP) findings have demonstrated that faces cueing a speaker’s identity were effective in increasing grammar processing of foreign accented speech, while the authors suggest that face information may have prepared listeners that the incoming speech may deviate from native pronunciations [20].

Regarding the role of language experience in accent intelligibility, research generally suggests that as exposure to different L2 accents increases, listeners’ linguistic representations become more flexible, and in turn, listeners can recognize and understand a larger variety of pronunciations [21]. However, this relationship is less straightforward for multilinguals, who may (or may not) share a native language with an L2 speaker and vary in the usage and age of acquisition of L2. For instance, after exposure to Japanese-accented English, multilinguals that reported a low usage of English had significantly worse comprehension ratings for Japanese-accented English speech than monolingual English listeners [22]. In contrast, multilinguals who reported regular usage of English had significantly better comprehension ratings for Japanese-accented English than monolingual English listeners. This suggests that the effects of increased exposure affect multilinguals more intensely, and the direction of its effects depends on the usage of L2. Relatedly, Kutlu et al. [23] investigated the influence of listeners’ language use on accent intelligibility by converting listeners’ self-reported daily language use into language entropy scores: a continuous, rather than discrete, measure of multilinguals’ language use [24]. Results from Kutlu et al. [23] suggest that listeners from a more linguistically diverse local (in this case, Montreal, QC, Canada) transcribed accented speech significantly more accurately than listeners from a less linguistically diverse local (in this case, Gainesville, FL, USA). Therefore, the balanced use of two or more languages may enhance the intelligibility of accented speech.

Similarly, research on the interlanguage intelligibility benefit, or the enhancement accent intelligibility resulting from speakers and listeners sharing the same L1 and L2, offers further nuance to the relationship between language exposure and accent intelligibility. For example, previous work suggests that multilingual listeners who share the same native language as the speaker will perform significantly better than monolinguals on intelligibility tasks if they acquired the L2 language at an early age but may perform significantly worse than monolinguals on intelligibility tasks if they acquired the L2 language after the age of 16 [25]. Moreover, when multilingual listeners do not share an L1 with the speaker, intelligibility findings are controversial, with some studies claiming this results in a mismatched speech intelligibility benefit [26] and other studies claiming this instead has detrimental effects on intelligibility [27].

In terms of sociolinguistic processing, less is known about bilingual participants. Broadly, numerous studies suggest that compared to monolingual children, bilingual children show less social preference for children who look like themselves and may hold less implicit bias against racialized groups [28,29]. Additionally, studies on bilinguals’ and monolinguals’ face discrimination abilities show evidence of bilinguals being more accurate in discriminating other-race faces compared to monolinguals, which is an effect that may be moderated by bilinguals’ proficiency of L2 independent of other-race exposure [30,31]. These studies demonstrate the saliency of linguistic background in processing race as a social cue.

Similar to the discrimination of outgroup-race faces, evidence suggests bilinguals may be better at discriminating different accents compared to monolinguals due to heightened sociolinguistic awareness. For instance, Evans and Tomé Lourido [32] found that while all children performed similarly in a comprehension task after listening to stories read in native, unfamiliar regional, or unfamiliar foreign accents, multilingual children outperformed monolingual children in categorizing the different accent types. To explain this finding, the authors proposed that compared to monolingual children, multilingual children are exposed to communities in which linguistic variants are socially meaningful. Navigating these communities arguably presents a greater demand for using linguistic information relative to more linguistically homogenous environments, and these demands may in turn lead to heightened sociolinguistic awareness in multilinguals [32] (p. 156). Taken altogether, these studies suggest that bilingual listeners may be more sensitive to phonetic differences between accents and more flexible in their consideration of speaker race when processing and identifying accented speech.

Finally, listener attitudes about non-native speakers have been shown to influence accent perception. For instance, Simon et al. [33] had native-speaking Dutch participants answer questions about their views on non-native Dutch speakers. Then, participants listened to, transcribed, and rated the comprehensibility and accentedness of sentences spoken by non-native Dutch speakers. The results showed that in general, listeners who held more positive attitudes toward non-native Dutch speakers rated the accent they heard as being more comprehensible and less strongly accented than listeners who held more negative attitudes toward non-native Dutch speakers. However, attitudes seemed to have no effect on intelligibility, as no significant correlations were found between listeners’ attitudes and listeners’ transcription scores. These results suggest that listener attitudes may weaken or enhance the comprehension of and perceived strength of accented speech, though its effects on intelligibility may be less straightforward.

The current study is a comprehensive investigation of bilingual and monolinguals sociolinguistic processing. The chief objective was to explore the impact of listener characteristics and race-based linguistic expectations on accent intelligibility and perceived accentedness. Mandarin accent intelligibility was tested in White English monolinguals and White English/Norwegian bilinguals. Norwegian was selected, as its speakers are predominantly White. Intelligibility was assessed between-subjects using a transcription task with Mandarin-accented English speech samples. Listener characteristics, including language use, language exposure, attitudes toward non-native speakers, and ability to identify Mandarin-accented English were assessed using a questionnaire developed specifically for this study. Items in this questionnaire consisted of questions handpicked and adapted from previous studies [33,34,35,36]. Notably, Maio and Kang [36] found that accent familiarity measures across the research field were two factored and therefore tapped into two separate underlying facets of accent familiarity. To account for this, the current study’s questionnaire used multiple methods to measure accent familiarity, including generic Likert scales, an audio-prompted accent identification task, and questions concerning contact and exposure, proficiency in Mandarin, proficiency in English, and age of language acquisition. Individual differences in age of language acquisition and usage of English and Mandarin were captured to account for possible moderation effects on multilingual listeners’ accent intelligibility [22,25]. Furthermore, following Kutlu et al. [23] and Simon et al. [33], listeners’ responses to language use and attitude items were used to calculate language entropy and average attitude scores, respectively. Broadly, in terms of listener characteristics, we predicted that language entropy, average attitude score, accent identification, and language background (monolingual versus bilingual) would all have significant main effects on transcription accuracy and accentedness rating. Finally, regarding the effects of race-based expectations, we predicted that transcription accuracy would significantly increase and accentedness rating would significantly decrease when Mandarin-accented English was paired with a White face compared to when it was paired with an East Asian face, which is in line with an exemplar model. Alternatively, aligning with the bias-based model, the opposite pattern of results for transcription accuracy and accentedness rating would emerge.

## 2. Materials and Methods

### 2.1. Participants

Two hundred and eighty American participants were recruited to complete this study using Prolific (https://www.prolific.com/, accessed on 9 April 2024). Participants were fluent in English and were over the age of 18. Participants were selectively recruited from Prolific based on their user demographic information. English monolinguals needed to self-report only being fluent in English and identify as White. English/Norwegian Bilinguals needed to identify as bilingual, list only English and Norwegian as languages they were fluent in and identify as White. Participants who disclosed any speech, hearing, or language disorders were excluded from the study. Relevant data of participants, including participant age, gender, current country of residence, nationality, and country of birth were collected via Prolific’s pre-collected user data and were linked to the data from our experiment and questionnaire. With regard to gender, 117 participants identified as women, 131 identified as men, and 7 identified as non-binary. Participants were on average 40.8 years old (SD = 12.86, range = 20–85).

### 2.2. Sample Size Determination

One hundred and forty participants were collected from each language background (monolingual and bilingual) and were sorted into one of two test conditions, creating four groups of 70 participants. McGowan [10] reported a 7% transcription accuracy difference between the congruent and incongruent face conditions. Power was computed for a similar effect size (7.5%) and for sample sizes ranging from 30 to 90 per condition. Power was computed as follows. For each sample size, 1000 datasets were simulated with accuracy values between 4 and 10 words for each of 60 utterances. Accuracy for the White face condition was set to 50%. Between participant and between utterance variability in the probability of a correct response as well as residual variability were estimated using data from a sample of 30 pilot participants. For each dataset, a generalized linear mixed model with a logit function was conducted. Accuracy was modeled as a function of condition (dummy coded, with one condition being the reference level), a by-participant random intercept, a by-utterance intercept and a by-utterance random slope. Power was computed as the percentage of significant models (for each sample size). Results of the power analysis show that if we assume an effect size of 7.5%, 70 participants per condition was needed to reach a power of 90% for this effect size.

### 2.3. Stimuli

#### 2.3.1. Auditory

Auditory stimuli consisted of 60 Mandarin-accented English sentences (e.g., “the whole sky was full of birds”) spoken by the same female speaker. Sentence files were embedded in multi-talker babble and equated in amplitude with an 8 dB signal-to-noise ratio. This STN ration was deemed suitable following piloting. Sentences ranged in length from 6 to 11 words long and were presented sequentially and in randomized order.

#### 2.3.2. Visual

**Figure 1:**
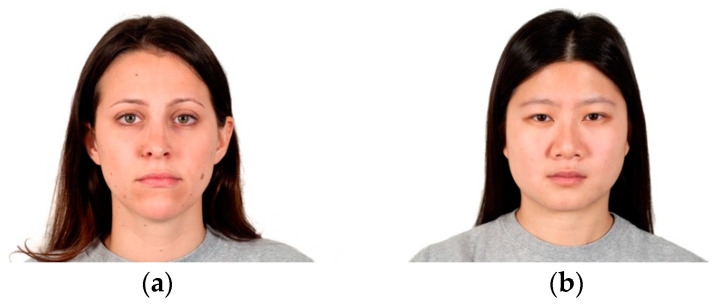
White face (**a**) and East Asian face (**b**) taken from the Chicago Face Database.

Visual stimuli consisted of two standardized photographs of faces taken from the Chicago Face Database [37]. The two photographs used consisted of a White face and an Asian face, and they were closely related in age, showed little to no emotional valence, and each face highly matched the desired ethnicity as suggested by ratings from norming data (Figure 1).

### 2.4. Procedure

Participants completed the study online on LabVanced software (Version 1.1.21, https://www.labvanced.com/, accessed on 9 April 2024). Participants were randomized into the White face and East Asian face condition. During the experiment, participants were told they would hear sentences and see the face of the speaker. Participants were told to adjust their audio volume as they saw fit. Unlike McGowan [10], participants were not told the language origins of the speaker in order to limit listeners’ expectations about the speaker to those created by the visual stimuli.

#### 2.4.1. Accent Transcription Task

Participants were then instructed to transcribe sentences that would be presented one at a time and played only once without repetition. Additionally, participants were informed that although speed did not matter, spelling did matter, so they must transcribe each sentence as accurately as possible. Sentence order was randomized across participants. Each trial began with 500 ms of blank noise with one of two visual stimuli, as seen in Figure 1. Then, an individual audio file played, and a text box appeared for participants to type their transcriptions. After they had finished transcribing, participants used the right arrow button to progress to the next trial.

#### 2.4.2. Post-Transcription Tasks

Following the transcription task, participants were asked to rate the strength of the accent heard in the previous task on scale of 1–7 (1 = no foreign accent, 7 = very heavy foreign accent). After rating the strength of the accent from the transcription task, participants completed an accent identification task. Following Miao and Kang [36] and Huang [38], the accent identification task functioned as an objective measure of familiarity with Mandarin-accented English to complement self-report measures. The task prompted participants to listen to one of the Mandarin-accented English sentences from the accent transcription task spoken by a different, male speaker, and they were told to identify the speaker’s native language or country of origin. A different speaker was chosen for the accent identification task to ensure the measure tapped into participants’ ability to detect Mandarin-accented English rather than encouraging participants to make their best guess based on the face shown in the accent transcription task. The accent identification task was followed by language attitude questions replicated from Simon et al. [33]. For language attitude questions, listeners were asked to indicate on a scale from 1 (strongly disagree) to 5 (strongly agree) how strongly they agreed with the following statements: “I could be friends with a non-native speaker who doesn’t speak English very well”; “conversations with non-native speakers of English often run smoothly”; “understanding non-native speakers of English requires a lot of effort”. These items functioned as a self-report measure of participants’ attitudes held toward non-native speakers. Then, using questions adapted from the Language History Questionnaire [34], bilingual listeners’ language use of English and Norwegian in home, work, school, friends, and overall contexts were elicited with the prompt “please rate the amount of time you actively use the following language(s) in [context] on a scale of 1–7 (1 = no usage at all, 7 = all the time).” Afterwards, participants completed Mandarin and English proficiency questions that were replicated from Miao and Kang [36] and Saito and Shintani [39]. Upon completion of the study, participants were debriefed and compensated with USD 4.50.

### 2.5. Analyses

#### 2.5.1. Coding

Transcriptions from the accent transcription task were coded to determine each participant’s mean transcription accuracy score. Transcription accuracy scores were calculated according to correctly identified words in each sentence. Participants were assigned a transcription score for each sentence, which was calculated as the proportion of correctly identified words over the total number of words in the sentence. Participant accuracy scores were averaged across sentences to determine each participant’s final mean transcription accuracy score for statistical analysis; these final mean participant accuracy scores were further averaged across the monolingual and bilingual participant groups.

For post-transcription tasks, participants’ open responses in the accent identification task were coded as correct (a score of 1) only if the response referred to China, Chinese, or Mandarin, following Huang [38]. All other responses were coded as incorrect (a score of 0). Coding revealed that 24.8% of monolingual listeners and 49.6% of bilingual listeners identified the accent correctly from the accent identification task. Scores across language attitude items were combined into a total score out of 15, wherein higher scores reflected more positive attitudes held toward non-native English speakers. The item “understanding non-native speakers of English requires a lot of effort” was reversed-scored to reflect this, while the other two items were scored normally.

#### 2.5.2. Language Entropy

Following Kutlu et al. [23], bilingual listeners’ language use was accounted for as a continuous rather than discrete variable using the languageEntropy package [24] in R (Version 4.3.1, https://www.r-project.org, accessed on 3 July 2024). First, bilingual listeners’ self-reported language use data were converted to proportions. These proportions were then used to calculate entropy values for each participant using the following entropy formula [40]:

$$H\;=\;-\sum_{i=1}^{n}P_{i}\log_{2}(P_{i})$$

This resulted in each participant receiving a value between 0 and 1 for each language context. A language entropy value of 1 indicated a completely balanced use of both Norwegian and English within a given context, whereas a language entropy value of 0 indicated the use of only one language within a given context. Following Gullifer and Titone [24], we conducted a Principal Component Analysis (PCA) by grouping correlated language entropy contexts to reduce the complexity of the dataset. We used varimax rotated components and used a scree plot of eigenvalues to select our final number of components. This resulted in two components, which will be referred to as C1 and C2 hereafter. Home, friends, and overall entropy loaded into C1 and explained 48.3% of the data. Work entropy, with some cross-loadings from friends and overall entropy, loaded into C2 and explained 31.8% of the data. A considerable number of participants (N = 38, 26.2% of the sample) left the school context blank, so this context was not included in our PCA (the individual varimax component loadings and scree plot are provided on OSF).

#### 2.5.3. Main Analyses

Data were preprocessed, analyzed, and plotted using R (Version 4.3.1, https://www.r-project.org, accessed on 3 July 2024) and jamovi (Version 2.3, https://www.jamovi.org/, accessed on 3 July 2024). Three generalized linear mixed effects models were fitted to the data using the lme4 package in R [41]. Depending on the model, fixed effects consisted of condition, language group (monolingual vs. bilingual), language entropy (language use) components, accent identification, language attitude score, and/or trial number (ranging from 1 to 60 depending on the order of sentence/trial presentation); participant identification (PID) and sentence were included as random effects for all three models with both random intercepts and random slopes. To ensure that the first estimate of the model (2-1) reflected the difference between the two conditions, effect coding and sliding contrasts were used with 1 = East Asian face, and 2 = White face. Average language attitude score and language entropy components were centered around their means. Scaled sum contrasts were used for the language group and accent identification variables, and trial number was standardized as a *z*-score. Follow-up tests were conducted with the emmeans package [42] and corrected pairwise comparisons with the Holm correction.

## 3. Results

### 3.1. Comparing Monolinguals and Bilinguals

#### 3.1.1. Accent Transcription Task

**Figure 2:**
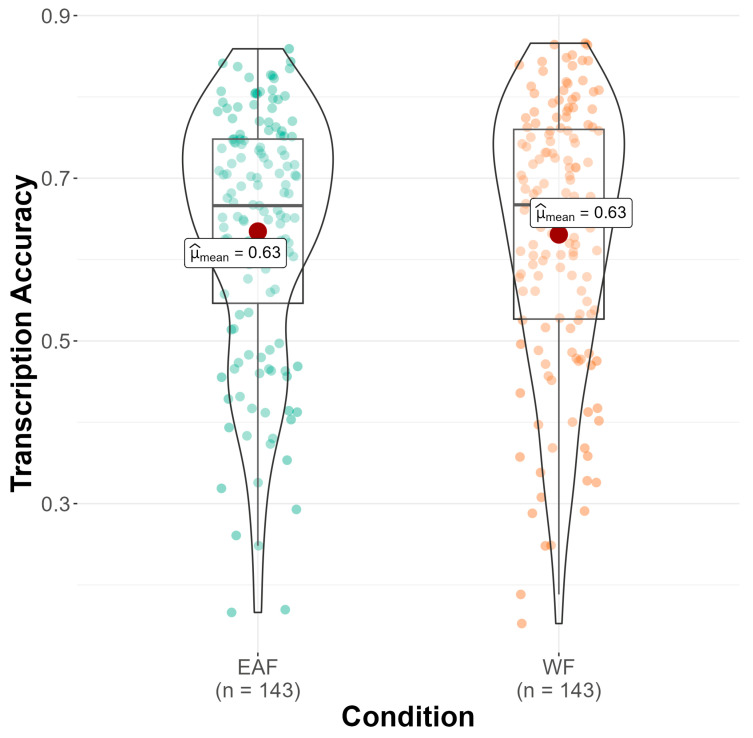
Transcription accuracy (*y*-axis) by condition (*x*-axis). Mean transcription accuracy indicated with a red dot.

First, we compare monolinguals’ and bilinguals’ performance in the accent intelligibility task using a linear mixed effects model (see OSF). For this model, condition, language group (monolingual vs. bilingual), accent identification, language attitude score, and trial number were included as fixed effects; participant identification (PID) and sentence were included as random effects with both random intercepts and random slopes. This model explained 33.6% of the variance in the bilingual and monolingual data and was the best fit when compared to other models. As seen in Figure 2, sentences paired with a White face were not transcribed differently than sentences paired with an East Asian face, *p* = 0.955, suggesting that the race of the speaker did not influence intelligibility. Furthermore, no main effect of language group was found, *p* = 0.176.

**Figure 3:**
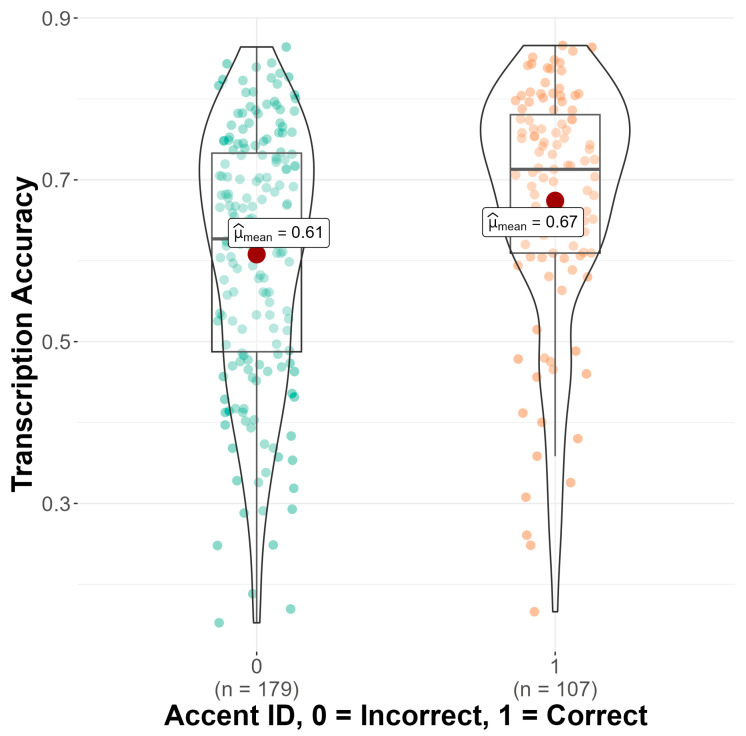
Transcription accuracy (*y*-axis) by accent ID (*x*-axis). Mean transcription accuracy indicated with a red dot.

**Figure 4:**
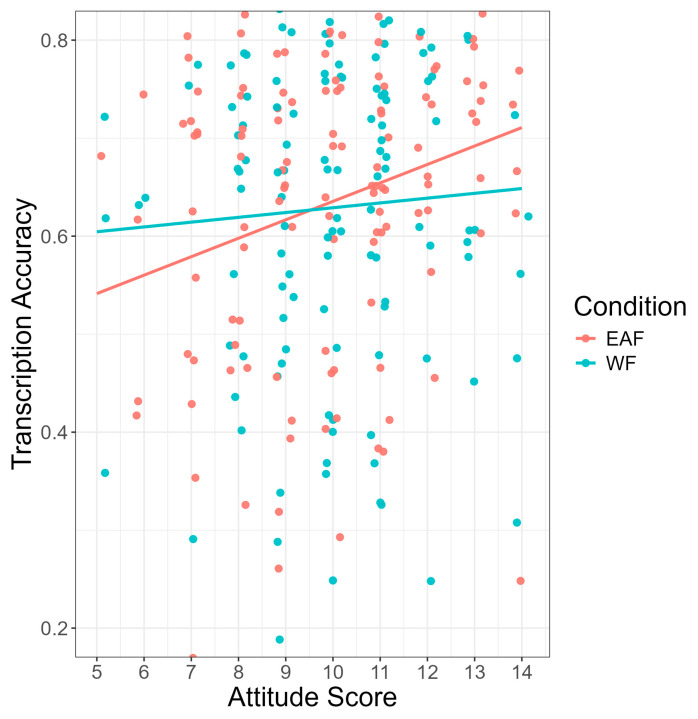
Transcription accuracy (*y*-axis) by attitude (*x*-axis). Average language attitude score had a range of 0–15 with higher scores reflecting more positive attitudes held toward non-native English speakers.

As seen in Figure 3, a significant main effect of accent identification was found (b = −0.21, SE = 0.05, z = −3.81, *p* < 0.001), such that monolingual and bilingual listeners who identified the accent correctly in the follow-up questionnaire transcribed sentences significantly more accurately (M = 0.67, SD = 0.15) than monolingual and bilingual listeners who did not identify the accent correctly (M = 0.61, SD = 0.17). Results also showed a significant main effect of language attitude score on transcription accuracy (b = 0.06, SE = 0.03, z = 2.28, *p* = 0.023), such that having more positive attitudes toward non-native speakers was associated with higher transcription scores (see Figure 4). Finally, there was a significant main effect of trial number (b = 0.14, SE = 0.01, z = 20.66, *p* < 0.001), suggesting that monolingual and bilingual listeners’ average transcription accuracy scores increased as the experiment progressed. No significant interaction effects between condition and language group, accent identification, attitude score, or trial number were found.

#### 3.1.2. Accentedness Ratings

**Figure 5:**
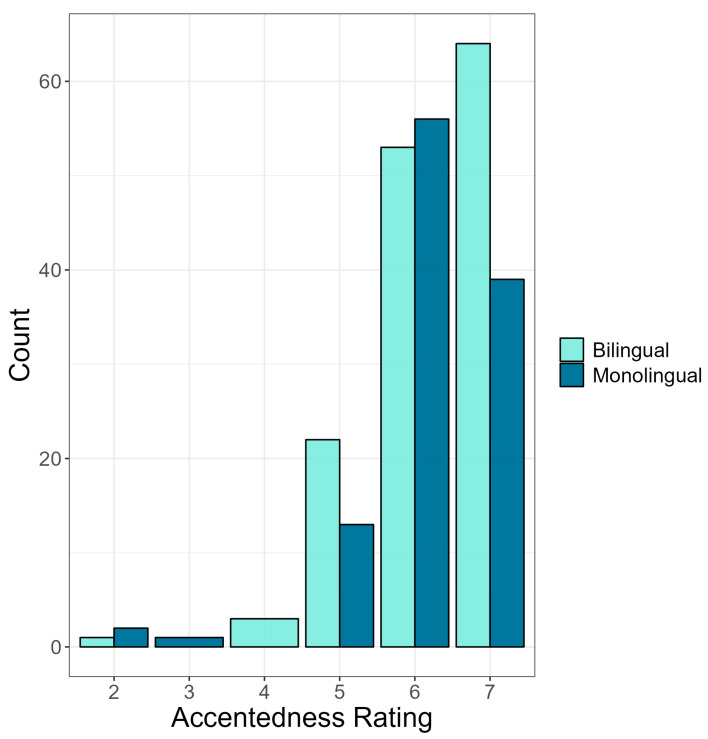
Accentedness ratings (*x*-axis) by language group (monolingual vs. bilingual). Accentendness rating was on a scale of 1–7 (1 = no foreign accent, 7 = very heavy foreign accent).

**Figure 6:**
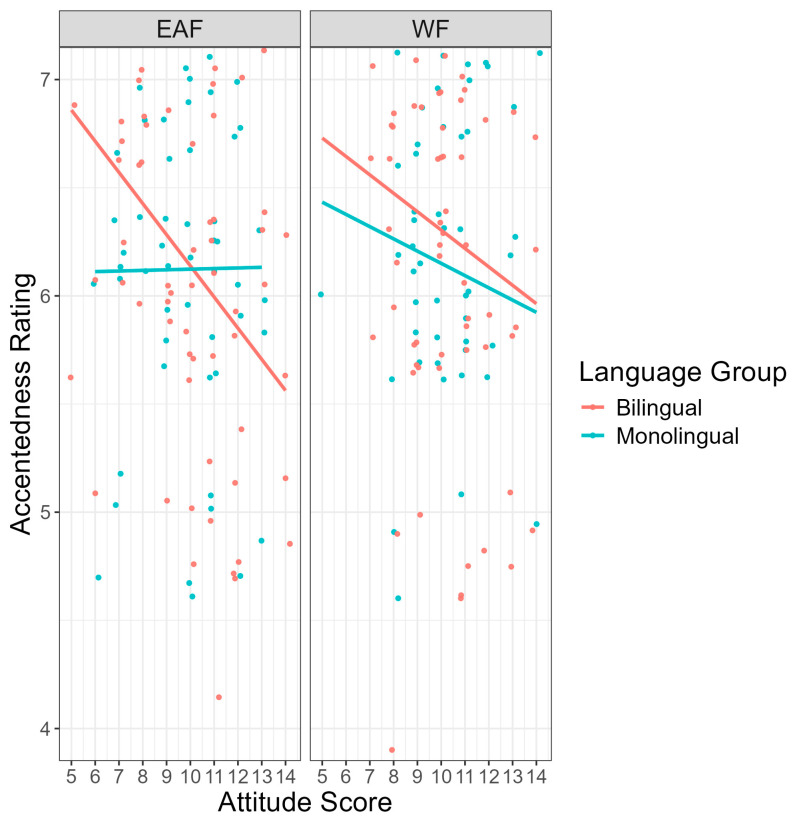
Accentedness ratings (*y*-axis) by attitude (*x*-axis), language group, and condition. East Asian face (EAF) condition pictured on the left; White face (WF) condition pictured on the right. Average language attitude score had a range of 0–15 with higher scores reflecting more positive attitudes held toward non-native English speakers.

Next, we compare monolinguals’ and bilinguals’ accentedness ratings using a cumulative link mixed model (see OSF). For this model, accentedness rating was the outcome variable, and condition, language group, accent identification, and language attitude score were entered as predictor variables; participant identification (PID) was entered as a random intercept. Random slopes were eliminated, as the model did not converge. Results showed no significant difference in accentedness rating between conditions, *p* = 0.778, suggesting that the race of the speaker did not influence perceptions of accentedness. However, as seen in Figure 5, the results did show that the accentedness rating differed significantly by language group (b = 1.62, SE = 0.65, z = 2.50, *p* = 0.012), such that bilingual listeners judged Mandarin-accented English as being more strongly accented (M = 6.22, SD = 0.87) than monolingual listeners (M = 6.14, SD = 0.90). Moreover, the results showed that accentedness rating differed significantly by language attitude score, (b = −0.18, SE = 0.06, z = −2.80, *p* = 0.005) such that more positive attitudes held toward non-native speakers were associated with lower accentedness ratings. In contrast, no significant main effect of accent identification was found, *p* = 0.072, suggesting that accent identification did not influence the perceived strength of the accent. Finally, as seen in Figure 6, a significant three-way interaction was found between condition, language group, and language attitude score (b = −0.20, SE = 0.08, z = −2.36, *p* = 0.018), suggesting that attitudes held toward non-native speakers influences the perceptions of accentedness differently depending on the race of the speaker and the language background of the listener. No other interaction effects between condition, language attitude score, language group, and accent identification reached significance.

### 3.2. Bilinguals

#### 3.2.1. Accent Transcription Task

Next, the role of language use and experience was analyzed in relation to bilinguals’ performance in the accent intelligibility task using a linear mixed effects model (see OSF). For this model, condition, accent identification, language attitude score, trial number, and language entropy components C1 and C2 were included as fixed effects with the same random effects as the previous linear mixed effects model. This model explained 32.5% of the variance in the bilingual data. Results showed no main effect of condition on transcription accuracy (b = 0.08, SE = 0.14, z = 0.54, *p* = 0.59), demonstrating that bilingual listeners transcribing scores in the White face condition (M = 0.626, SD = 0.16) did not differ statistically from their scores in the East Asian face condition (M = 0.625, SD = 0.16). However, the results did show a significant main effect of accent identification (b = −0.22, SE = 0.07, z = −3.31, *p* = 0.001), such that bilingual listeners who identified the accent correctly in the follow-up questionnaire transcribed significantly more accurately (M = 0.67, SD = 0.15) than bilingual listeners who did not identify the accent correctly (M = 0.58, SD = 0.16). The results also showed a main effect of trial number (b = 0.14, SE = 0.01, z = 15.1, *p* < 0.001). The results showed no main effects of language entropy components C1 and C2 as well as no main effect of language attitude score.

**Figure 7:**
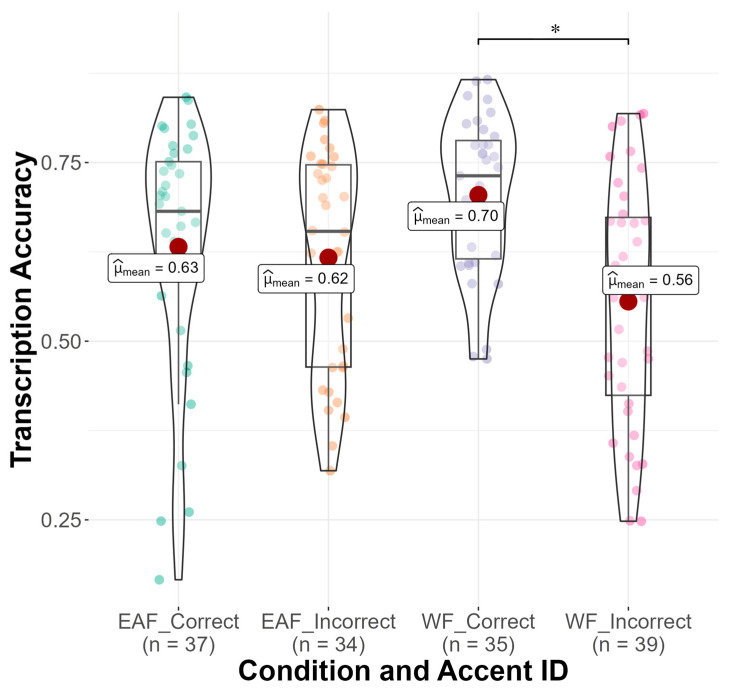
Transcription accuracy (*y*-axis) by condition and accent ID (*x*-axis). Asterisk (*) indicates significant interaction.

**Figure 8:**
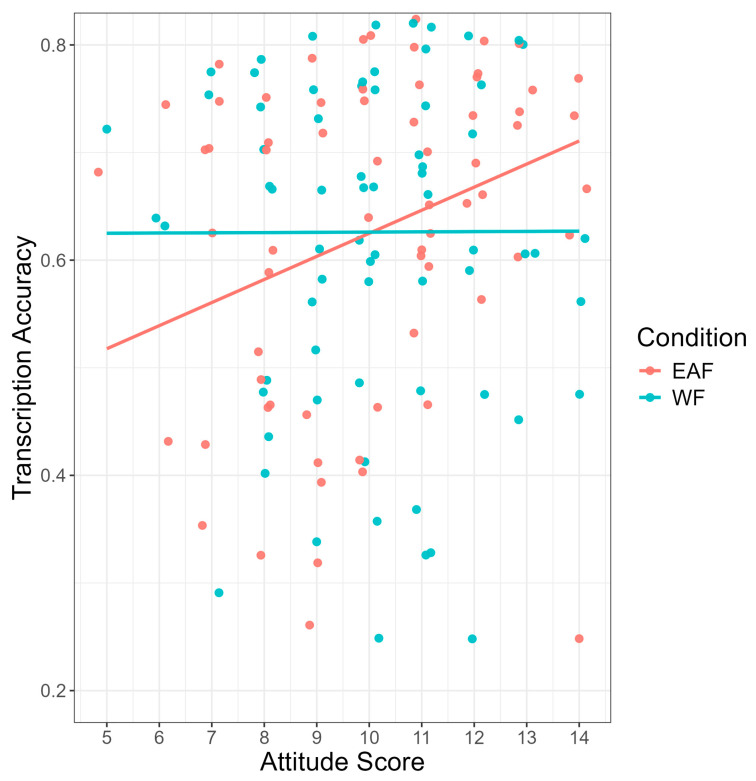
Transcription accuracy (*y*-axis) by attitude (*x*-axis) and condition. The East Asian face (EAF) condition is pictured in red, and White face (WF) condition is pictured in blue. Average language attitude score had a range of 0–15 with higher scores reflecting more positive attitudes held toward non-native English speakers.

The results showed two significant interaction effects. Firstly, the results showed a significant interaction between condition and accent identification (b = −0.45, SE = 0.14, z = −3.34, *p* = 0.001). As seen in Figure 7, in the White face condition, bilingual participants who identified the accent correctly transcribed sentences significantly more accurately than participants who incorrectly identified the accent (b = −0.79, SE = 0.19, z = −4.23, *p* < 0.001). This effect was not found for bilinguals in the East Asian face condition. Finally, the results showed a significant interaction between condition and language attitude score (b = −0.16, SE = 0.07, z = −2.43, *p* = 0.015). As seen in Figure 8, in the East Asian face condition, as average language attitude scores increase (i.e., more positive attitudes held toward non-native speakers), transcription accuracy also increases. No other significant interaction effects were found.

#### 3.2.2. Accentedness Ratings

**Figure 9:**
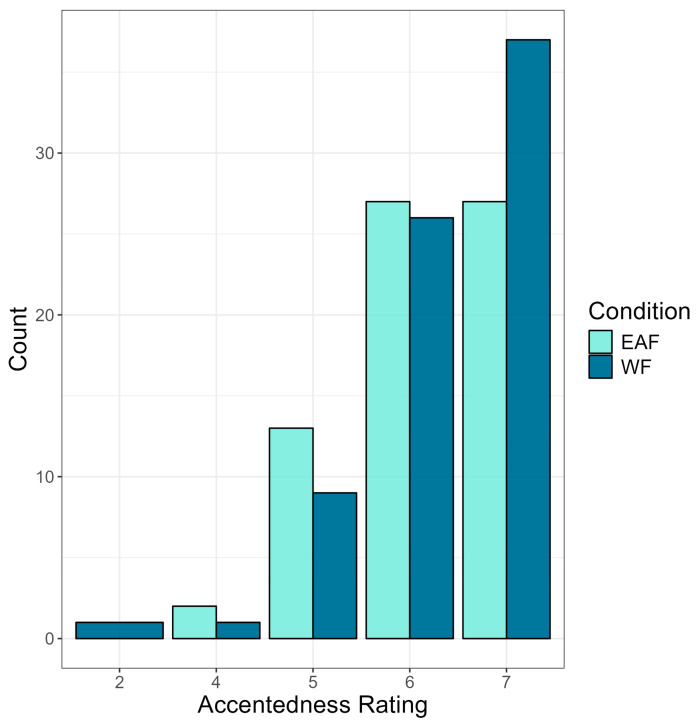
Accentedness rating (*x*-axis) by condition. Accentendness rating was on a scale of 1–7 (1 = no foreign accent, 7 = very heavy foreign accent).

Next, we investigated the role of bilinguals’ language use and experience of the perceived strength of an accent using a cumulative link mixed model (see OSF). For this model, accentedness rating was the outcome variable, and condition, language entropy component C2, and language attitude score were entered as predictor variables. The model failed to converge with the inclusion of language entropy component C1 and accent identification. Random effects included were the same as the previous cumulative link model. As seen in Figure 9, the results showed no significant difference in accentedness ratings between conditions, *p* = 0.142, again suggesting that speaker race does not influence perceived accentedness. However, a significant main effect of C2 on accentedness rating was found (b = −0.35, SE = 0.17, z = −2.02, *p* = 0.044), such that as C2 increases, accentedness rating decreases. This suggests that a balanced language use of English and Norwegian in work, friends, and overall contexts was associated with lower accentedness ratings. Finally, a significant main effect of language attitude score on accentedness rating was also found (b = −0.27, SE = 0.08, z = −3.18, *p* = 0.001). These results suggest that bilingual listeners’ language use and attitudes held toward non-native speakers influence the perceived strength of an accent. No significant interaction effects were found between condition, C2, and language attitude score.

### 3.3. Monolinguals

#### 3.3.1. Accent Transcription Task

**Figure 10:**
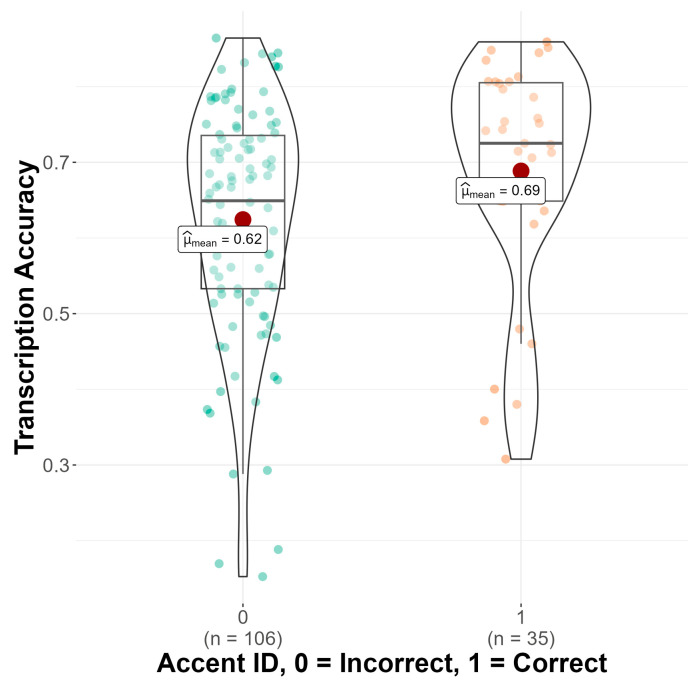
Transcription accuracy (*y*-axis) by accent ID (*x*-axis). Mean transcription accuracy indicated with a red dot.

Next, we delved deeper into monolinguals’ performance in the accent intelligibility task using a linear mixed effects model (see OSF). For this model, condition, accent identification, and trial number were included as fixed effects with the same random effects as the other linear mixed effects models. Language attitude score could not be added to this model, as 30 participants (21%) did not complete the language attitude items in the linguistic experience questionnaire. This model explained 35.8% of the variance in the monolingual data. Results showed that sentences paired with a White face were not transcribed differently than sentences paired with an East Asian face, *p* = 0.813 with monolingual listeners transcribing sentences in the White face condition (M = 0.636, SD = 0.17) and sentences in the East Asian face condition (M = 0.644, SD = 0.14) almost exactly the same. Again, this suggests a lack of influence of speaker race on accent intelligibility. However, as seen in Figure 10, the results revealed a significant main effect of accent identification (b = −0.18, SE = 0.08, z = −2.20, *p* = 0.028). Inspection of transcription accuracy means by accent identification reveals that monolingual listeners transcribed significantly more accurately when they were able to correctly identify the accent in the follow-up questionnaire (M = 0.69, SD = 0.15) compared to when they were not able to identify the accent correctly (M = 0.62, SD = 0.15). Finally, the results showed a significant main effect of trial number (b = 0.18, SE = 0.01, z = 13.0, *p* < 0.001). All interaction effects failed to reach significance.

#### 3.3.2. Accentedness Ratings

**Figure 11:**
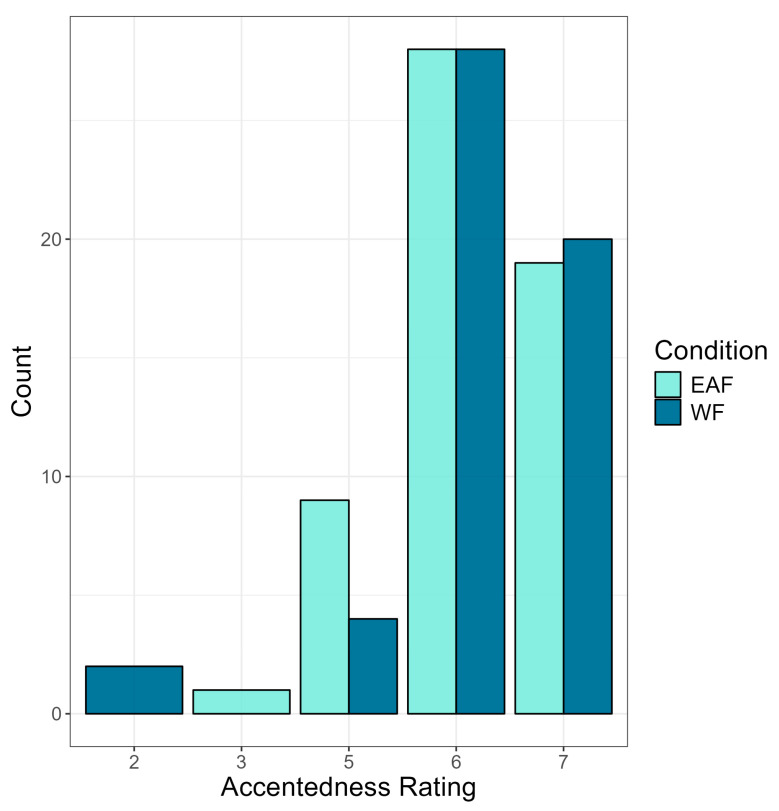
Accentedness rating by condition. Accentendness rating was on a scale of 1–7 (1 = no foreign accent, 7 = very heavy foreign accent).

Finally, a cumulative link mixed model was used to examine monolinguals’ accentedness perceptions more closely (see OSF). For this final model, accentedness rating was the outcome variable, and condition, accent identification, and language attitude score were entered as predictor variables. Random effects included were the same as the previous cumulative link models. As seen in Figure 11, the results showed no significant difference in accentedness ratings between conditions, *p* = 0.605, further suggesting the lack of influence of speaker race on accentedness perception. The results also showed no significant main effect of accent identification on accentedness rating, *p* = 0.535, as well as no significant difference in accentedness rating by language attitude score, *p* = 0.824. Finally, no significant interaction effects were found between condition and accent identification, *p* = 0.850, or between condition and language attitude score, *p* = 0.570.

## 4. Discussion

The current study sought to better understand the combined effects of speaker race and language experience on accent intelligibility in White monolingual and bilingual listeners. The findings demonstrated that neither transcription accuracy nor accentedness ratings were influenced by speaker race for monolingual or bilingual participants. This contrasts with most previous research that suggests pairing different faces with accented speech has weakening or enhancing effects on transcription accuracy as well as changing the perceived strength of the accent [10,13,15,16,17,23,43].

Unlike McGowan [10], the current study provided no information about the speaker’s language background to limit listeners’ expectations about the speaker to those created by the visual stimuli. Without explicitly priming the listener’s expectations about the speaker’s language background, speaker race alone may not have yielded strong expectations about the speaker’s accent. However, past studies have found a significant effect of race on intelligibility and accentedness without providing information about the source of the presented speaker’s accent; but critically, these studies had within-participants designs that presented multiple pairings of different faces and accents, likely increasing the saliency of an association between the two [23,43]. In contrast, for between-participant designs, the face’s saliency as a speaker cue may reduce over time. Offering some support for this explanation, Paladino and Mazzurega [44] found that incongruent presentations of accent and race created a parallel activation of in-group and out-group categorizations and, after repeated exposure, accent had a larger effect on categorization than race. Therefore, unless attention is purposely drawn to the race of a speaker, accent and non-native speaker status are likely far more salient speaker cues than speaker race.

While no main effect of condition was found, our results do suggest that intelligibility and accentedness are affected by participants’ language experience. When analyzing bilinguals, the results show that it was only in the White condition that sentences were transcribed significantly more accurately when the Mandarin accent was correctly identified. In contrast, this interaction effect was not found when monolinguals were analyzed separately. These findings raise the perplexing question of why accent identification did not enhance transcription accuracy when Mandarin-accented English was paired with an East Asian face and why this interaction effect was only present for bilingual listeners. Firstly, as evidenced by coding results for the accent identification task, only 24.8% (N = 35) of monolingual listeners were able to correctly identify Mandarin-accented English compared to 49.6% of bilingual listeners (N = 72). Therefore, it is likely there were not enough monolingual listeners who identified the accent correctly to observe an interaction between condition and accent identification for this group. Secondly, it is possible that, as predicted by the bias-based model, pairing an East Asian face with accented speech activated biases and stereotypes in the listener, and this prevented any enhancement effects of correct accent identification. These results may be further contextualized by the influence of attitudes on transcription scores. To this end, bilingual listeners’ language attitude scores significantly interacted with condition such that listeners’ who are more biased toward non-native speakers were worse at transcribing the accented speech than those who held more positive attitudes toward non-native speakers but only when it was an East Asian face paired with the accent. These results align with Kang and Rubin [18], who found that negative listener attitudes were associated with significantly lower listener comprehension ratings of standard American English speech when it was paired with an East Asian face but not when the same speech was paired with a White face. Altogether, current and past findings suggest that bilinguals’ biases play a role in their accent intelligibility. That is, accent identification and attitudes toward non-native speakers may affect the perception of accented speech differently depending on the speakers’ race.

In terms of accentedness ratings, we found that bilingual listeners tended to judge Mandarin-accented English as being more accented than monolingual listeners, which is consistent with findings that language experience influences perceived accent strength [21,22,39]. Additionally, listeners’ accentedness judgements were influenced by speaker race and attitudes toward non-native speakers differently depending on whether the listener was bilingual or monolingual. That is, bilinguals’ attitudes seemed to influence accentedness ratings strongly in the East Asian condition, whereas monolinguals’ attitudes did not. It must be noted that drawing any definite conclusions on the influence of monolinguals’ attitudes on accentedness ratings may not be appropriate given the large number of missing responses to attitudinal items in the monolingual group (N = 30). Furthermore, past research has suggested that generational differences can modulate the effects of listener attitudes on the perception of accented speech [45]. Therefore, future research is warranted to further investigate the combined effects of listener attitudes and language background on the perceived strength of an accent.

At first blush, the overall finding that bilinguals’ sociolinguistic processing is more in line with a bias-based model than monolinguals may come as a surprise. By all accounts, bilingual listeners may be better at adapting to the needs of their communication partners [46], may exhibit less implicit racial bias [29,47,48,49], and may have increased contact with outgroup members and decreased outgroup negativity, depending on the communities they are part of [28,49]. However, while bilinguals may hold less implicit bias against racialized groups, this does not necessarily mean that they are less likely to hold race-based linguistic expectations. In fact, because bilinguals are exposed to communities in which linguistic variants are socially meaningful, social variants may become more salient to them. This is particularly true in the case of accent processing, as bilinguals have been shown to struggle more in linguistic tasks such as word recognition when listening to accented speech as compared to monolinguals [27,50,51]. Navigating communities with more linguistic variability presents a more cognitively demanding environment in which listeners must contend with more variability, and this demand may lead to heightened sociolinguistic awareness in multilinguals [32]. To this end, bilinguals are more accurate in discriminating other-race faces compared to monolinguals [30,31], and bilingual children are better at discriminating and categorizing accents than monolingual children [32]. Also, it is critical to recall that the bilinguals in this study are White English–Norwegian bilinguals. Thus, although they may have experienced more linguistic diversity than the monolingual participants, their relative exposure to Mandarin speakers is comparable to that of the monolingual speakers. This homogenous group of monolinguals was intentionally recruited to control for language experience and to match monolingual and bilinguals in terms of race and experience with race. These results should not necessarily be generalized to all bilinguals.

The present study is the first step in determining the degree to which language experience influences sociolinguistic development. However, it is not without limitations, such as a lack of consideration for listener age. Furthermore, the current study’s follow-up questionnaire relied almost entirely on self-report Likert scales, which is subject to issues such as social desirability bias and constraints on self-knowledge [52]. Furthermore, all our participants were recruited from the U.S with most bilinguals’ self-report responses having indicated a relatively large number of years spent in the U.S., high English proficiency, and a relatively young age of acquisition of English (see OSF for descriptive statistics). Thus, we cannot generalize our results to include all non-native speakers and bilingual listeners, as it is possible that speech samples from speakers with different native languages and the inclusion of bilinguals proficient in other languages may have yielded different results.

## 5. Conclusions

In conclusion, our research contributes to the growing body of research in sociolinguistic processing, offering a unique comparison of monolingual and bilinguals’ intelligibility performance and accentedness ratings and how the two may interact with speaker race, accent identification, language use, and language attitudes. Our results speak to the subjectivity of accent perception, having critical implications across a variety of domains, such as the language assessment for the determination of origin (LADO) and the wider field of forensic linguistics, the development of curriculum material for L2 learners, and relationships between native-speaking employers and non-native speaking employees [6,53]. Our results should give researchers the confidence to extend our findings to more contexts, such as investigating a more diverse sample of bilinguals, in order to further our understanding of accent perception and its critical implications for an increasingly globalized world [5].

## Data Availability

The original data presented in the study are openly available in Open Science Framework at https://osf.io/pnk42/?view_only=1e2201bc27e743dab7694f1ff0bb6319 (accessed on 27 August 2024).
